# Dr. Bailey’s Case of Hepatic Colic

**Published:** 1843-03-18

**Authors:** J. R. Bailey

**Affiliations:** Russellville, (Ky.)


### THE MEDICAL EXAMINER,

### And Retrospect of the Medical Sciences.

Vol. VI.] PHILADELPHIA, SATURDAY, MARCH 18, 1843. [No. 5.

RUSSELLVILLE, (Ky.) March 5th, 1843.

To the Editor of the Medical Examiner.

*Sir*,—On reading the lecture of Prof. Chomel on "Hepatic Colic," published in the Medical Examiner of the 11th February, I was induced to refer to the subjoined notes of an interesting case, which happened in my practice some years since. They are imperfect in many respects, as you will see, having been taken with no view to publication, but only to keep the more important points of the case in recollection. Should you deem them of sufficient interest for the columns of your journal, they are at your disposition.

I remain yours, respectfully,

J. R. BAILEY, M. D.

—

Mr. F. T——s, a middle aged man, had, up to the 22d December, 1838, suffered from occasional severe attacks of colic for the last eight years, from which he generally recovered in about a week or so. He always noticed that his alvine dejections, for some days previous to these attacks, were of a whitish, clayey appearance, and invariably assumed a very dark colour, with purging, as the symptoms of colic declined.

He likewise observed that the attacks were more frequent latterly—having come on once a month, or once every fortnight; whereas, formerly, there was an interval of two, three, or even six months. Although they had varied much in severity, yet he could not say that they were more violent as they became more frequent. He had at various times taken some Thomsonian remedies, which, though they greatly relieved the pain at the commencement of the attack, did not sensibly shorten its duration. He had rarely felt well for the last six months; complained of a dull pain in the region of the liver, with loss of appetite, and occasional nausea. A few weeks before this time he had suffered from a severe fit of colic, followed by a diarrhœa, which, persisting longer than usual, rendered him very weak.

At the above date he was also labouring under irregular intermittent fever. The nausea had become constant, and his countenance presented a sallow hue. A mercurial treatment, (short, however, of ptyalism,) was adopted, which checked almost instantly the intermittent fever; however, at the end of two weeks, there was no perceptible improvement in other respects. I considered ptyalism necessary; and for that purpose directed calomel (two gr.) regularly every six hours, and his right hypochondrium to be rubbed with an ointment containing calomel and tartarised antimony, until the gums became sore. This was effected in a few days, and a copious crop of pustules brought out. A decided amendment was soon evident. His improvement was rapid, and his appetite became even ravenous. He considered himself entirely well; but his wife still noticed an occasional flushing of his cheeks.

Whilst Mr. T—— was busily engaged in his farming operations, he was attacked with colic on 16th March, 1839. The attack was lighter than usual. His alvine evacuations had some days before assumed the clayey character; and although I had warned him of the necessity of taking medicine, immediately on perceiving such a state of things, yet he had neglected my admonitions, thinking "a man with so good an appetite could be in no danger." The following pills were ordered, one every fourth hour.

| | |
|---|---|
| ℞. Hydrarg. Proto-chlorid. | ʒss. |
| Ipecacuanhæ, | gr. vi. |
| Syrupi Simpl. | q. s. |
| Ft. pil. vi. | |

In twenty hours large bilious evacuations were produced, and he was very much relieved. No salivation resulting from this course, and the patient being averse to undergo this process, I was induced to try the nitric acid. He commenced with six drops, and increased it to ten, but with no sign of improvement; (he, however, only used it four or five days.) On the 22d, being called in haste, I found him worse than at any previous time. I gave an active emetic of ipecacuanha, with no apparent benefit, followed by a mercurial cathartic, which relieved the urgent symptoms, and I proceeded to induce ptyalism in the same manner as formerly.

The relief was such, that on 4th April, while his gums were yet tender, he ventured abroad (contrary to orders, however,) to superintend his farm. On 6th he felt somewhat worse. Soreness of the gums subsided, on the supervention of a severe rigor, on the evening of 7th. The rigor continued for two hours, with excruciating pain in the stomach and right hypochondrium. A burning heat of skin followed, of about two hours duration, succeeded immediately by a second and even a third rigor, each following the course of the first. During these twelve hours he suffered the greatest agony. After the third rigor I gave the sulphate of quinia, in conjunction with the calomel he was again taking. The rigors ceased; but still he complained much of his side, and particularly of his stomach. Dr. Withers saw the patient with me on 8th, and, at his suggestion, an emetic of ipecac. was administered, the calomel increased from four to eight grains every four hours, and given in combination with carb. iron, &c. Although not salivated, his dejections in forty-eight hours became bilious. The colour of the evacuations at this time was ashey; they were likewise foamy; a tinge of greenish yellow was barely perceptible. (Bile, having the same characters, was found, on the post-mortem examination, in the stomach and excretory ducts of the liver.) He was much relieved of his sufferings, but the nausea continued as distressing as at any time, with occasional pain in the stomach. No complaint of the right hypochondriac region. He sunk gradually—indeed, I may say rapidly, and died on 21st April, sixteen days after his last attack.

Although several remedies were prescribed at various times, yet nothing seemed to relieve him except calomel and counter-irritation by tart. emet. and a blister was once applied to his side.

During the latter part of his sickness his pulse was exceedingly feeble and small, and not increased in frequency.

*Autopsy thirteen hours after death. (Time limited.)*

Body much emaciated.

Head not examined.

Lungs and heart sound in every respect.

Stomach nearly empty, containing only a few ounces of viscid matter coloured with bile, as mentioned above. Several cicatrices were apparent on the mucous coat—the largest about the size of a 25 cent piece. The whole of the great curvature was strongly injected with blood, and the posterior portion of the curvature was of a dark brown colour. Mucous lining injected throughout.

Bowels healthy, except that part of the transverse arch of the colon which lay in contact with the stomach. This part was, as the stomach, strongly injected.

Liver of a natural colour, except the posterior edge of the right lobe, which presented a brownish red colour; and a small portion of the anterior edge of the same lobe was of a dark venous hue.

On cutting into the right lobe, commencing on the convex surface, and proceeding in the direction of the portæ, I noticed on one side of the incision a marked difference of colour. Fluctuation was evident to the touch. I opened it, and a fluid foamy bile, as described above, made its exit, and showed the sac to be a distended portion of one of the excretory ducts—the bile being prevented from passing by a small gall-stone. It was of a jet black colour, easily reduced to powder by rubbing between the fingers, to which it communicated a deep, though bright yellow colour, as it did also to water. On examining my incision more particularly, I found I had opened several similar cavities, though smaller, but had overlooked the gall-stones, they being very small, some not larger than a pin-head—all very black, the slightest scratch being sufficient to reveal the bright yellow colour. Continuing the incision, I found, as it approached the portæ, these bodies became larger and more numerous; two (and in one instance three) being found in a sac. Each of the three was as large, if not larger, than a pea. The largest met with was about three-fourths of an inch in transverse, and one and three-fourths in longitudinal diameter. It was firmly imbedded in the substance of the liver, filling up completely the portion of duct in which it was situated, and extending into two branches, which, at that point, united to form one. There were all gradations of size, from this to the small ones mentioned.

No gall-stones could be found in any part of the left lobe, though they might be found in all parts of the right.

The smaller gall-stones were uniformly constituted throughout, excepting a more porous texture in the centre. The largest one, (which I have preserved,) consists of a central nucleus, with several concentric layers of deposite, marked by a shade of variation in the colour. Its internal texture was not so bright as in the smaller ones.

The gall-bladder was *entirely empty*, and of a *blanched* appearance.

